# Selection of Suitable Reference Genes for Analysis of Salivary Transcriptome in Non-Syndromic Autistic Male Children

**DOI:** 10.3390/ijms17101711

**Published:** 2016-10-12

**Authors:** Yasin Panahi, Fahimeh Salasar Moghaddam, Zahra Ghasemi, Mandana Hadi Jafari, Reza Shervin Badv, Mohamad Reza Eskandari, Mehrdad Pedram

**Affiliations:** 1 Department of Genetics and Molecular Medicine, School of Medicine, Zanjan University of Medical Sciences (ZUMS), Zanjan 45139-56111, Iran; panahi.y@zums.ac.ir (Y.P.); mandana.hj@gmail.com (M.H.J.); 2 Department of Medical Biotechnology, School of Medicine, Zanjan University of Medical Sciences (ZUMS), Zanjan 45139-56111, Iran; f.moghaddam91@gmail.com (F.S.M.); zahra.ghasemi77@gmail.com (Z.G.); 3 Department of Pediatric Neurology, School of Medicine, Tehran University of Medical Sciences (TUMS), Tehran 14176-13151, Iran; badv@sina.tums.ac.ir; 4 Children’s Medical Center, Pediatric Center of Excellence, Tehran University of Medical Sciences (TUMS), Tehran 14176-13151, Iran; 5 Metrowest CNS Research Center, Natick, MA 01760, USA; dr.eskandari@gmail.com; 6 Department of Psychiatry, School of Medicine, Zanjan University of Medical Sciences (ZUMS), Zanjan 45139-56111, Iran

**Keywords:** childhood autism, non-syndromic, transcriptome, saliva, reverse transcriptase quantitative real-time PCR (RT-qPCR), housekeeping genes (HKGs), reference gene, stability of expression, geNorm, NormFinder

## Abstract

Childhood autism is a severe form of complex genetically heterogeneous and behaviorally defined set of neurodevelopmental diseases, collectively termed as autism spectrum disorders (ASD). Reverse transcriptase quantitative real-time PCR (RT-qPCR) is a highly sensitive technique for transcriptome analysis, and it has been frequently used in ASD gene expression studies. However, normalization to stably expressed reference gene(s) is necessary to validate any alteration reported at the mRNA level for target genes. The main goal of the present study was to find the most stable reference genes in the salivary transcriptome for RT-qPCR analysis in non-syndromic male childhood autism. Saliva samples were obtained from nine drug naïve non-syndromic male children with autism and also sex-, age-, and location-matched healthy controls using the RNA-stabilizer kit from DNA Genotek. A systematic two-phased measurement of whole saliva mRNA levels for eight common housekeeping genes (HKGs) was carried out by RT-qPCR, and the stability of expression for each candidate gene was analyzed using two specialized algorithms, geNorm and NormFinder, in parallel. Our analysis shows that while the frequently used HKG *ACTB* is not a suitable reference gene, the combination of *GAPDH* and *YWHAZ* could be recommended for normalization of RT-qPCR analysis of salivary transcriptome in non-syndromic autistic male children.

## 1. Introduction

Autism spectrum disorders (ASD) are serious, lifelong pervasive neurodevelopmental disorders characterized by impairments in reciprocal social interaction, including verbal and non-verbal communication, and also repetitive stereotyped behavioral patterns [1]. Childhood autism, which is the most severe form of ASD with an early life onset, is typically diagnosed between the ages of 2 and 3 years [2] and a male to female ratio of about 4:1 [3]. There is no doubt that genetic components play a key role in the pathogenesis of ASD with a high heritability index. However, the genes that have been associated with ASD only relate to only a small portion of the cases [4].

Due to the wide range of genetic heterogeneity reported in ASD, it is quite possible that different causes and various underlying molecular mechanisms may lead to a common set of changes in the brain resulting in a similar behavioral profile [5,6]. Nonetheless, analysis of the transcriptome, as an intermediate phenotype, still could provide valuable insights for investigation of such complex cases. Careful comparison of the gene expression profiles in autistic patients with normal subjects could provide important clues for the development of autism and also the molecular mechanisms and crossroads involved. Reverse transcriptase quantitative real-time PCR (RT-qPCR) is a highly sensitive technique for investigation of gene expression profile, and it is also used for confirmation of microarray results [7,8]. However, accurate analysis of gene expression with RT-qPCR requires proper normalization to stably expressed internal control or reference gene(s) [9]. An important caveat to consider for selection of reference genes, which are typically selected from housekeeping genes (HKGs), is that expression of HKGs could vary significantly under different biological conditions including disease states, age, sex, drug treatment. Furthermore, the stability of expression for a particular HKG could also be dependent upon the tissue type/source used for RNA isolation and experimental design [10,11]. Therefore, selection of appropriate reference genes for a complex neurodevelopmental disorder such as autism requires very careful design and planning.

A review of the literature shows that no single gene or particular set of genes has been used consistently as reference in gene expression studies in ASD, using either the brain or blood as the main RNA source (Table 1) [12,13,14,15,16,17,18,19,20,21,22,23,24,25,26,27,28,29,30,31,32,33,34]. Surprisingly, as we will further outline in the discussion, almost none of these studies have embarked on a systematic validation of the reference gene(s) prior to the start of the experiment. In the present study, we: (1) propose using saliva as a readily available biofluid and an alternative noninvasive sampling source for transcriptome analysis in childhood autism; and (2) provide a systematic evaluation for the expression levels of eight commonly used HKGs in whole saliva samples in drug naïve non-syndromic autistic male children, as a highly important yet defined subset of ASD patients, and healthy sex-, age-, and location-matched controls.

## 2. Results

### 2.1. Selection of Candidate Reference Genes and Initial Screening

The present study was prompted by an earlier experiment, during which saliva samples were obtained from five lab members and β-actin gene (*ACTB*) mRNA levels were measured for RNA quality analysis. It should be noted that *ACTB* has been used previously by other investigators for RNA quality and transcriptome analysis by RT-qPCR in saliva [35,36,37,38], and that it also has been used as a common internal control for gene expression studies in ASD (Table 1). Interestingly, while *ACTB* *Cq* values were stable in the salivary transcriptome derived from the lab members (i.e., adults), the *Cq* values varied when examined in five samples (one autistic and four healthy control subjects) taken from children (Table S1).

There were limited amounts of saliva samples taken from our clinical groups, collected within a two-year period, available for future studies. Thus, in order to systematically evaluate potential reference genes for normalization of gene expression levels in whole saliva collected from patients with non-syndromic childhood autism and the healthy controls, a two-phase analysis was performed. In the first phase, for initial screening, mRNAs levels of eight candidate HKGs (Table 2) were examined by RT-qPCR using equal amounts of RNA templates extracted from four saliva samples: two samples taken from autistic patients, and two from healthy controls. The RT-qPCR procedures were designed and performed in line with the Minimum Information for Publication of Quantitative Real-Time PCR Experiments (MIQE) guidelines [10]. The mean *Cq* values ± SD (*n* = 2–4) for each candidate gene are presented on Table 3 (see Table S2 for detailed individual average *Cq* values of replicate qPCR reactions ± SD).

To analyze the stability of expression for the candidate HKGs, the *Cq* values of each data set were exported to a Microsoft Office Excel sheet and then imported into the geNorm (qbasePLUS software v2.2, Biogazelle, Ghent, Belgium) and also NormFinder (Molecular Diagnostic Labrotory, Department of Clinical Biochemistry Molecular Medicine, Aarhus University Hospital, Skejby, Aarhus, Denmark) software. GeNorm, which is a strong algorithm for small sample sizes and also the most widely used algorithm to determine the most stable reference gene, calculates an expression normalization factor for each sample set by geometric averaging of a number of user-defined candidate reference genes. For each candidate gene, the geNorm algorithm determines an internal control stability measure (M), which is defined as the average pairwise variation of a particular gene compared with all other test genes. The lowest M value indicates the most stable gene [40]. By contrast, NormFinder that is also a visual basic application for Microsoft Excel focuses on the expression variation of each gene with the least intra- and inter-group values in a model-based approach. In addition to giving a direct measure for the estimated expression variation, the NormFinder algorithm evaluates the systematic error introduced when using the HKG of interest as internal control [41].

**Figure 1:**
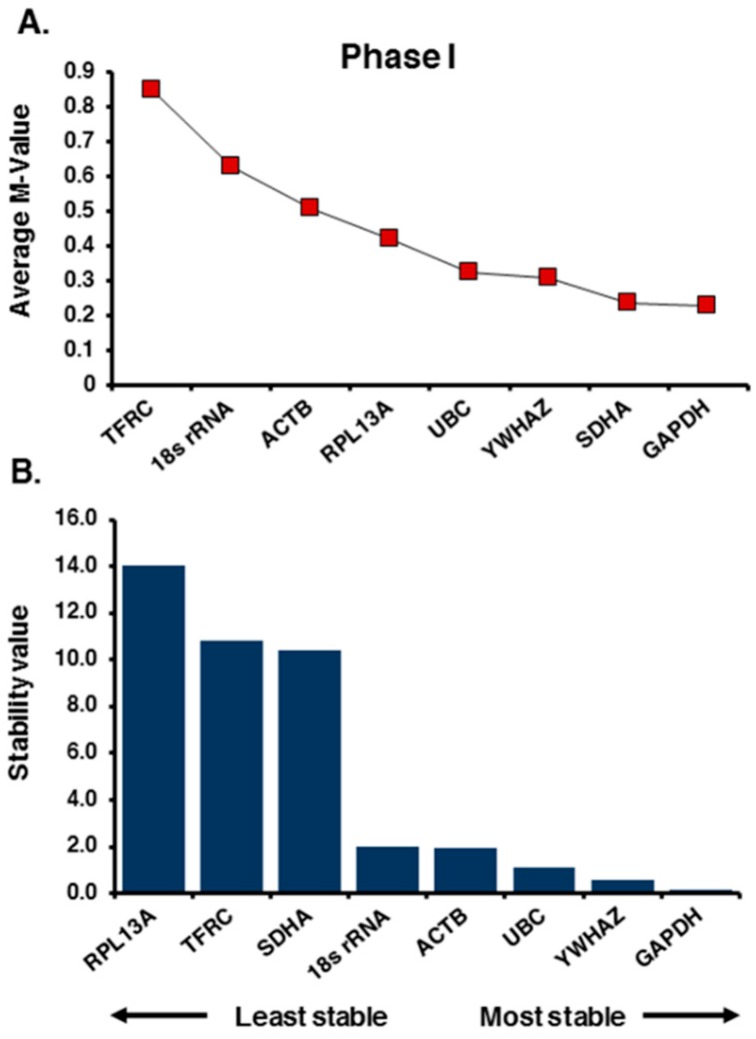
Expression stability analysis of the candidate reference genes in phase I. The stability of expression for 8 housekeeping genes tested in saliva is ranked (left to right) based on the least stable to the most stable gene by: (**A**) geNorm; and (**B**) NormFinder algorithms.

According to the geNorm algorithm, the M values below the cut-off value of 1.5 (the recommended threshold) are acceptable in the selection of internal controls for normalization in RT-qPCR [40]. As it can be seen from Figure 1A, the data output from geNorm for the first phase of evaluation indicated that glyceraldehyde-3-phosphate dehydrogenase gene (*GAPDH*) was the best HKG in our panel of candidate reference genes followed by succinate dehydrogenase complex subunit A, flavoprotein (*SDHA*), tyrosine 3 monooxygenase activation protein, zeta polypeptide (*YWHAZ*), and ubiquitin C (*UBC*). *ACTB*, a traditionally common reference gene in normalization of RT-qPCR data in ASD studies (Table 1) and salivary transcriptome analysis [35,37,38], was ranked among the least stable genes. The number of reference genes needed for optimal normalization was determined by geNorm based on the average pairwise variation (V*n*/*n* + 1) below the recommended cut-off point value of 0.15. At this phase, the geNorm output indicated that the combination of the best three reference genes would be sufficient for normalization of RT-qPCR data (Figure S1).

The ranking of the best candidate genes by NormFinder algorithm in phase I was somewhat similar to the geNorm output. *GAPDH*, *YWHAZ*, and *UBC* were ranked as the best candidates, respectively (Figure 1B). However, *SDHA* was demoted to the 6th rank, while *ACTB* was promoted two steps from low to the intermediate ranking. It should be noted that, in phase I, the clinical identifier value was not included in the NormFinder analysis due to the small number of samples, and thus analysis was performed as a single clinical group. In both algorithms, two of the candidate genes, encoding for ribosomal protein L13a (RPL13A) and transferrin receptor (TFRC), were ranked as the least stable HKGs based on their high variation of relative expression values.

### 2.2. Expression Profiling and Validation of Candidate Reference Genes

**Figure 2:**
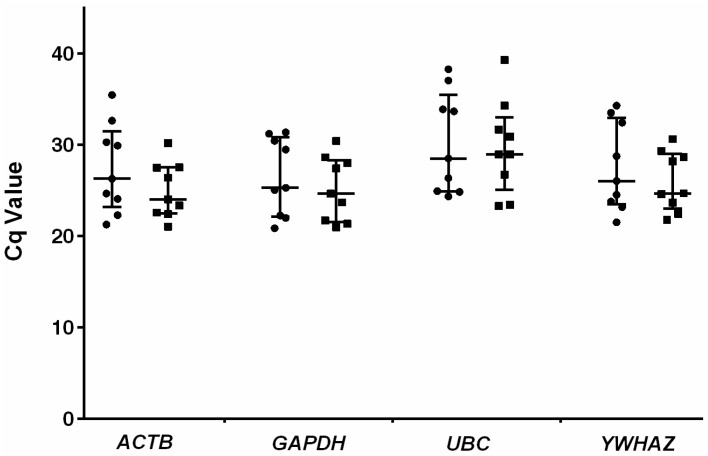
Scatter plot representations of the *Cq* values measured for the final four candidate genes. Filled squares and circles represent the control and autistic groups, respectively. The vertical bars indicate the median and interquartile range. The bottom and top of each bar represent the 25th and 75th percentiles, respectively, and the line bisecting each bar shows the median *Cq* value.

Three of the candidate HKGs that had the best scores in phase I based on both geNorm and NormFinder algorithms (*GAPDH*, *YWHAZ*, and *UBC*) were selected for the second phase of analysis. Despite its low-intermediate ranking in phase I, *ACTB* was also taken for further analysis because of its common usage as an internal control HKG in saliva [35,38,42] and ASD studies (Table 1). In this phase, the expression levels of the final four candidate HKGs were examined in saliva samples from a panel of nine autistic and healthy boys (average age: 47.6 ± 21.9 vs. 41.2 ± 26.7 months, respectively). The amplification efficiency for each gene and linear regression coefficient (R2) values were calculated by performing standard curves (Table 4). The scatter plot representations of the *Cq* values recorded for the final four candidate HKGs in the autistic and healthy clinical groups are shown in Figure 2 (Table S3 for detailed average *Cq* values for replicate qPCR reactions ±SD).

**Figure 3:**
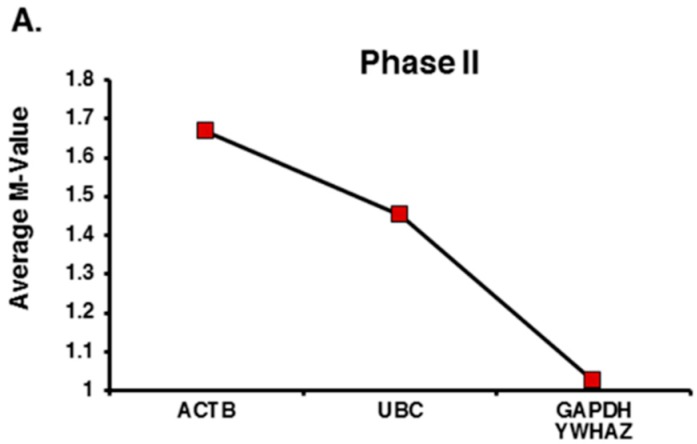

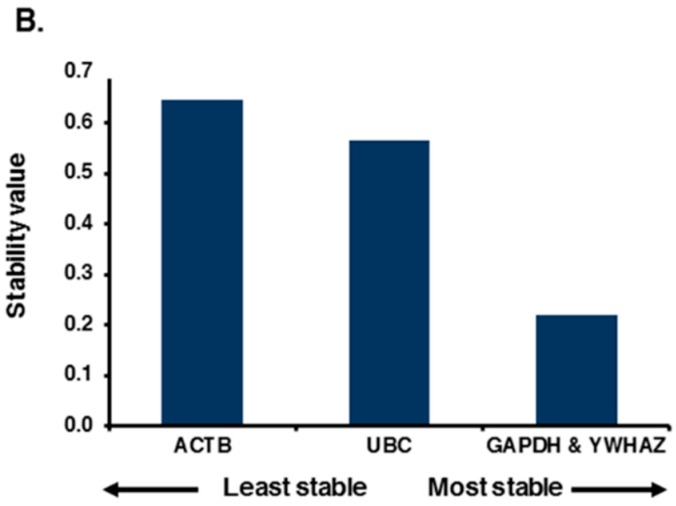
Expression stability analysis for the final four candidate reference genes in phase II. Expression of each candidate gene was examined in a total of 18 saliva samples (nine with childhood Autism and nine healthy controls) ranked compared with the rest by (**A**) geNorm and (**B**) NormFinder algorithms based on their stability of expression values; when the clinical group’s identifier was included in the NormFinder analysis, *GAPDH* and *YWHAZ* were selected as the best genes combined, in agreement with the geNorm pair-wise ranking.

Noticeably, geNorm once again ranked *GAPDH* as the most stable HKG, and it ranked *ACTB* as the least stable gene (*M* values: 1.29 vs. 1.84, respectively). However, after the pairwise analysis, the combination of *GAPDH* and *YWHAZ* was selected as the best pair with an *M*-value of 1.02, followed by *UBC* and *ACTB* with *M*-values of 1.45 vs. 1.67, respectively (Figure 3A). An optimal number for reference genes was not determined by geNorm algorithm because the *V*-value was higher than recommended threshold (0.15). Similar to the geNorm analysis, NormFinder evaluated *GAPDH* as the most stable HKG, followed by *YWHAZ*, *UBC*, and *ACTB* (Stability values: 0.35, 0.60, 1.18, and 1.40, respectively). However, when the clinical subgroups were included in the analysis, the *GAPDH* and *YWHAZ* set was recommended as the best combination of two genes by NormFinder, followed by *UBC* (ranked third) and *ACTB*, ranked as the least stable gene once again (Figure 3B).

### 2.3. Relative Expression Levels of Candidate Reference Genes in Autistic vs. Healthy Subjects

**Figure 4:**
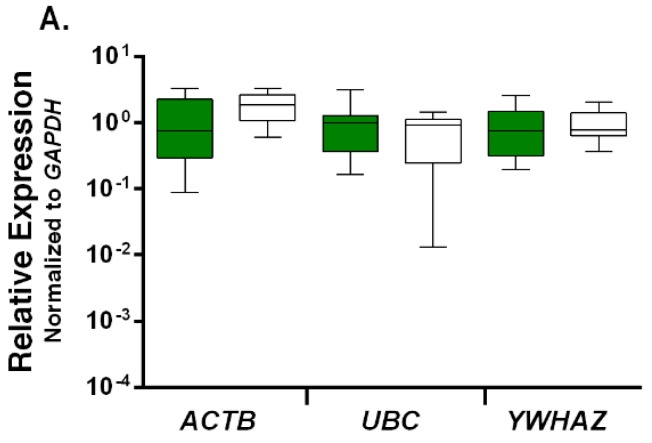

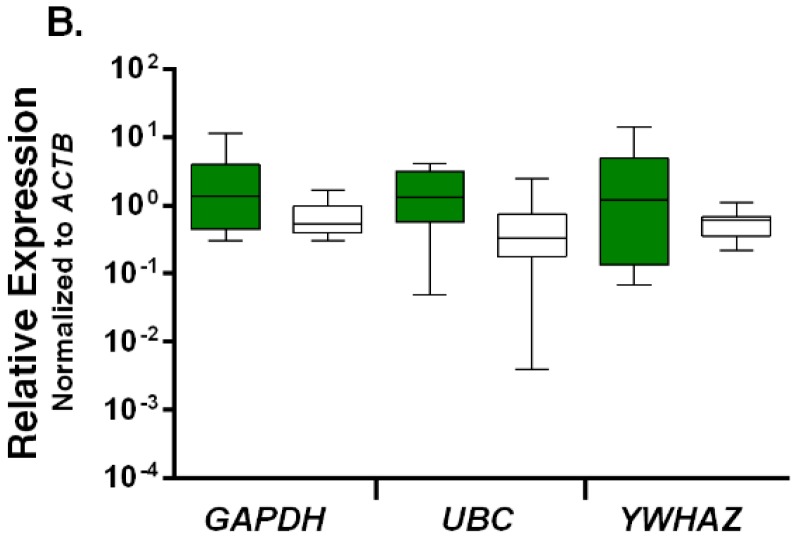
Comparison of the relative expression levels of candidate reference genes between the two groups. Colored and white boxes represent the autistic and control groups, respectively. The relative expression level for each gene was calculated by using either: (**A**) the *GAPDH* (glyceraldehyde-3-phosphate dehydrogenase); or (**B**) ACTB (β-actin) gene as the reference. Statistical analysis comparing the expression levels are done with Mann-Whitney *U* test (two-tailed), and the data are presented as the mean and interquartile range, with vertical bars representing the total range (**B**). *p*-values: (**A**) *ACTB* (*p* = 0.26), *UBC* (*p* = 0.45), *YWHAZ* (*p* = 0.82); and (**B**) *GAPDH* (*p* = 0.26), *UBC* (*p* = 0.03), *YWHAZ* (*p* = 0.26).

Using *GAPDH*, the most stable HKG ranked by both geNorm and NormFinder algorithms, as reference, and employing an improved 2^−Δ*C*t^ method [43] (by taking into account the amplification efficiencies), the relative expression levels of other three genes were compared in the autistic children vs. the control group (Figure 4A). The results demonstrate that although *ACTB* shows a higher degree of variation compared with *UBC* and *YWHAZ* (*p*-values: 0.26, 0.45 and 0.82, respectively), as expected, there are no significant differences in the expression levels of any of these HKGs between the two clinical groups. By contrast, when *ACTB*, which was ranked as the least stable, was used as reference, the expression levels of *GAPDH*, *UBC*, and *YWHAZ* did not follow a normal distribution pattern, and in particular, the *UBC* expression levels and median values were significantly different (*p* = 0.03) between the autistic and control groups (Figure 4B).

The above observations are in support of the ranking validity of the candidate reference genes by geNorm and NormFinder algorithms, and they clearly demonstrate how selection of an inappropriate HKG as reference can bias interpretation of RT-qPCR data. Interestingly, examination of the *Cq* values obtained for the candidate HKGs using a third statistical algorithm applet, called BestKeeper [44], produced a similar ranking for the candidate reference genes, in particular for Phase II (Table S4). It should be noted that the BestKeeper algorithm works by calculating the SD for each gene of interest and Pearson’s coefficient of correlation for each pair of the candidate genes. Thus, it would be best suited for our Phase II analysis, as BestKeeper cannot provide significant *p*-values for small sample sets (Table S4A,B). Nonetheless, based on the intra-variations for each candidate gene, the Phase I ranking by BestKeeper is not that far off from those produced by geNorm and NormFinder.

## 3. Discussion

To our knowledge, this is the first systematic evaluation of HKGs as reference for RT-qPCR analysis of salivary transcriptome in patients with autism reported so far. ASD encompass a heterogeneous set of complex and pervasive behaviorally-defined neurodevelopmental disorders, which brings up the possibility of different causes leading to similar brain deficits and behavioral patterns. In fact, there is a strong consensus on the involvement of various genetic and environmental components in the etiology of ASD [1,6]. Nonetheless, because of the high heritability reported in ASD, it should be quite plausible to search for a “common signature” at the level of gene expression profile. Thus, analysis of the transcriptome, as an intermediate phenotype for investigation, could provide insights into the molecular mechanism/s involved [5]. However, despite enormous number of research and concerted efforts at the international level during the past decade, as evident on Table 1, there is still an incoherent picture of a common gene expression profile signature/s for ASD, and the underlying molecular mechanisms are poorly understood.

A recent highly in depth meta-analysis of ASD gene expression data, taken from 12 independent studies in blood and brain tissues, by Ch’ng et al. conclude that constructing a somewhat consistent transcriptome signature might be possible in the studies using a small number of brain samples. By contrast, the outcomes of the studies using blood cells are highly heterogeneous and rather inconsistent [5]. As pointed out by the investigators, part of the inconsistency in these studies could be due to the variation in their inclusion and exclusion criteria, which include variations in the scope of the ASD patients studied, differences in gender and age groups, and differences in sampling and experimental procedures. A review of the information presented on Table 1 supports the notion of such variations in different ASD studies. Another important factor to note, however, is the inconsistency in the use of reference genes in different studies, even in cases where the type of tissue/cells used for transcriptome analysis were the same or very similar.

Selection of appropriate reference gene(s) plays an essential part in proper normalization of RT-qPCR assays and comparison of gene expression profiles between different samples and clinical groups. It is also essential that validity and stability of expression for the candidate or intended internal control HKG/s is experimentally examined for particular source/s of RNA, disease status, and experimental design prior to the start of each experiment [10,40,41]. Surprisingly, a careful examination of the ASD gene expression studies listed on Table 1, however, reveals that the HKGs used as internal controls were not systematically evaluated for their stability of expression for the particular tissue/cell type, and biological and experimental conditions. Perhaps, the only one exception on that list is the case report study by Griesi-Oliveira et al. using dental pulp stem cells for their main RNA source. Aside from the fact that the particular female patient in this study suffered from a craniofacial dysmorphism in addition to ASD phenotype, it should be noted that because of the very small and restricted sample size (only one patient and two controls from the same family), validation of the reference genes in this study has a very low power. It is also interesting to note that even with such a small and restricted sample size, when the investigators used the geNorm applet for selection of the reference genes, they ended up with a set of four HKGs in order to generate a normalization factor [30]. The study by Nardone et al., using postmortem brain tissues, used a set of four HKGs based on a geNorm evaluation of candidate reference genes by a separate group of investigators on suicide subjects [34,45]. Finally, the study by Garbett et al., using a small set (six; four male and two female subjects) of postmortem brain tissue samples, selected only one HKG, *ACTB*, for validation of their microarray results based on the previous works by the same lab [18] on small numbers of postmortem brain samples from schizophrenic and epileptic patients [46,47].

An ideal reference gene should maintain a stable mRNA level in all sample groups under different experimental conditions. It might not be possible to find a “universal” reference gene or set of genes in different tissues for various biological and experimental conditions. However, it is quite feasible to find the most appropriate HKGs for normalization of gene expression profiling if one limits the type and number of tissues tested under a well-defined experimental setting [40,41]. As mentioned several times already, ASD encompass a heterogeneous and complex set of neurodevelopmental disorders. In the present study, in an effort to limit the number of confounders and heterogeneity, we confined the scope of the ASD subjects to drug naïve non-syndromic autistic male children and age-, and sex-matched healthy children picked from the same neighborhoods as the patients (average ages: 47.6 ± 21.9 vs. 41.2 ± 26.7 months, respectively). Considering the severity of childhood autism and also the high ratio of male children affected, the autistic patients used in this study represent a highly important yet defined subset of ASD patients. We also used saliva as an alternative “noninvasive” sampling source for RNA instead of the blood. As the most available and portable biofluid, saliva contains a wealth of information regarding the physiological states of the body and is a good indicator of the plasma levels of hormones, drugs, immune system, and neurological conditions [48,49]. In recent years, saliva has been used a valid source of biomarkers and transcriptome analysis for a number of systemic human diseases including sleep disorder [38] and breast cancer [50].

Saliva has also been used as a DNA source for epigenetic analysis in bipolar disorder and schizophrenia [51], for genetic analysis and investigation of the impact of nutritional intake and environmental toxins in ASD [52]. Most recently, Frank Middleton and colleagues used the salivary microRNA profiles for identification of children with ASD [53]. Here, we present the first systematic evaluation of HKGs in the salivary transcriptome to be used for normalization of RT-qPCR data in ASD research. Based on our analysis of gene expression stability using geNorm and NormFinder algorithms in parallel, *GAPDH* was determined as the most stable HKG to be used as an internal control for salivary transcriptome analysis and/or validation of microarray results by RT-qPCR in male childhood autism. By contrast, *ACTB* was evaluated as unsuitable to be used as a reference gene. It should be noted, however, that *GAPDH* does not appear to be stable enough to be used as a single reference gene for normalization of RT-qPCR data in this setting. Both geNorm and Normfinder analyses recommend combination of *GAPDH* and *YWHAZ* as the best option. However, the V value given by the geNorm algorithm in our second phase of study is above the ideal 0.15. A simple interpretation of the results from geNorm’s point of view would entails using all four candidate genes as internal controls. However, considering the high variation of *ACTB* expression, and significantly low levels of *UBC* expression between the autistic and control samples (Figure S1 and Figure 4), it would not be advisable to add either of them to the *GAPDH* and *YWHAZ* combination.

The poor evaluation of *ACTB* as a reference gene in saliva samples, in a sharp contrast to *GAPDH*, initially came to us as a surprise. However, a careful review of the literature revealed this to be a well-supported outcome by various lines of evidence. While *ACTB* was traditionally used as a reference gene in saliva [38], even pioneer labs specializing in saliva work have recently switched to using *GAPDH* instead [50,54]. In contrast to this notion, in a recent report by Pandit and colleagues on a high-yield RNA-extraction protocol for saliva, the investigators used *ACTB* in combination with the saliva-specific histatin 3 gene (*HTN3*) for validation of the quality and quantity of their isolated RNA samples. They also used *ACTB* alone as their reference HKG for analysis of 2 cell lines and a cohort of cancer patients and controls [35]. However, a review of the *Cq* values listed for *ACTB* in their Table 1, shows a very high variation in the *Cq* values (18 Cq differences for all samples, and 13 Cq differences within the clinical samples only) for *ACTB*, making it unsuitable to be used as a reference gene. Furthermore, it should be kept in mind that the saliva samples taken in our study were obtained from young children (average age for both groups: 44.4 ± 23.9 months) that are going through a highly significant growth period. A key developmental and cytoskeletal protein coding gene like *ACTB* may not be suited as a reference gene for this age period. In support of this line of reasoning, a recent investigation of HKGs in leukocyte subpopulations from children by Yu and colleagues found that *GAPDH* had the most stable expression in children (five samples: 1, 3, 6, 9, and 12 years old). By contrast, the ACTB protein levels were inconsistent. The investigators reported that *ACTB* mRNA levels were not significantly different across their samples and propose a difference at the level of post-translational regulation. However, there is no mention of the *ACTB* *Cq* values/variation provided in the article [55].

In contrast to our findings indicating *ACTB* as an unsuitable internal control for RT-qPCR analysis in childhood autism, the study by Garbett and colleagues gives *ACTB* a high praise as a reference gene for transcriptome analysis in postmortem brain tissue samples from ASD patients [18]. Aside from the fact that we have used a different source for RNA, a close inspection of the report by Garbett et al. reveals some interesting points. The authors provide several reasons for why they chose *ACTB* as the reference gene for RT-qPCR verification of their microarray results, including that it had been previously established as a stable HKG in the literature (referring to a study by Chen et al. on the primary human skin fibroblasts [56]); and in two previous studies on human postmortem brain tissues by the same lab, one in subjects with schizophrenia [46] and one in epilepsy [47]. Aside from the fact that we have used a different source of RNA in the present study compared with the studies noted above, the reasoning outlined by Garbett et al. for selection of their internal control HKG is not convincing. The fact that *ACTB* has been previously used as an endogenous reference gene in primary human skin fibroblasts (a different tissue type), and that it has been used in the brain tissue samples in schizophrenia and epilepsy (different disease types), are not valid justifications for using *ACTB* as a reference gene in brain tissue samples for ASD studies. Two recent systematic evaluations of HKGs, with both studies including *ACTB* and *GAPDH*, for RT-qPCR in postmortem human brain tissues came up with entirely different sets of reference genes. While Silberberg and colleagues found *TFRC* and *RPLP0* as the most stable reference genes in schizophrenia and bipolar disorders [57], Penna and colleagues found CYC1 and EIF4A2 as the best reference genes in Alzheimer’s disease [58]. Finally, it is interesting to note that a review of the reference genes used in ASD studies shows a noticeable shift from *ACTB* to *GAPDH* during the past 15 years (Table 1).

## 4. Materials and Methods

### 4.1. Subjects and Sampling Procedure

Participants recruited to this study included nine drug naïve non-syndromic male children diagnosed with autism (47.6 ± 21.9 months) and nine age-, gender-, and location-matched typically developing healthy controls (41.2 ± 26.7 months). Children with autism were diagnosed according to the Diagnostic and Statistical Manual of Mental Disorders fourth edition, Text Revised (DSM-IV-TR) criteria by two qualified clinicians, a child neurologist and a child and adolescent psychiatrist. The diagnosis of autism was confirmed by Autism Diagnostic Interview-Revised (ADI-R), which is a standardized semi-structured diagnostic algorithm for autism based on the definitions set by DSM-IV and International Statistical Classification of Diseases and Related Health Problems 10th Revision (ICD-10) [59,60]. Exclusion criteria for patients included any other medical disorder and/or diagnosis including significant neurological problems such as depression, epileptic seizures, and genetic syndromes such as tuberous sclerosis, Angelman, and Fragile-X. Sex-matched healthy control children were picked from the same neighborhood locations as the autistic subjects. All healthy controls were screened by the Strengths and Difficulties Questionnaire (SDQ) [61]. The study was conducted in accordance with the Declaration of Helsinki, and it had the approval of the ZUMS Ethics Committee (ZUMS.REC.1392 97; 13 October 2013). All subjects had signed informed consent, provided by their parents, for inclusion before participating in the study.

Two mL of whole saliva was collected from each child who was able to provide sputum into the Oragene RNA collection kit RE-100 (DNA Genotek Inc., Ottawa, ON, Canada). Saliva sampling was supervised and collected by trained personnel according to the instructions provided in the collection kit. In the case of children unable to spit voluntarily, sampling was done by a 2-mL syringe from under the tongues and gingival crevices, mainly with the help of the participating parents and/or caregivers. Briefly, children were recommended to refrain from eating and drinking for 1 h prior to sampling and also wash their mouths with drinking water at least 15 min before providing a sample. Once collected, the tube containing the sample was covered by placing the cap securely and inverting the container vigorously by hand vortexing for approximately 10 s, in order to allow the saliva to mix well with the Oragene RNA solution. Samples were then stored at −20 °C and subsequently transferred to the central laboratory at the Zanjan University of Medical Science (ZUMS) for further processing.

### 4.2. Total RNA Preparation and cDNA Synthesis

Total RNA was extracted from whole saliva samples using a combination of two protocols: Oragene RNA collection kit RE-100 (DNA Genotek Inc., Ottawa, ON, Canada) and RNX-Plus solution (SinaClone BioScience, Tehran, Iran) with some modifications to the manufacturers’ instructions. Briefly, the saliva collected in the Oragene RNA collection kit was incubated in a water bath at 50 °C for 1 h. A 500-µL aliquot was then transferred into a 1.5-mL microcentrifuge tube and incubated in a water bath at 90 °C for 15 min. Twenty µL of the Neutralizer solution was added to the sample and vortexed for a few seconds, incubated on ice for 10 min, and centrifuged at 15,000× *g* for 3 min. The clear supernatant was then transferred into a new microcentrifuge tube, 2 volumes of cold 95% ethanol was added to the sample and mixed vigorously by hand. The sample was incubated at −20 °C for 30 min followed by centrifugation at 15,000× *g* for 3 min. The supernatant was discarded and the pellet was dissolved in 750 µL of RNX-Plus solution (SinaClone BioScience, Tehran, Iran) by vortexing, followed by incubation at room temperature for 5 min. A 200-μL volume of Chloroform was added to the solution and centrifuged for 15 min at 12,000× *g*. The upper phase was then transferred to a new microcentrifuge tube and an equal volume of isopropanol was added into the tube. The mixture was centrifuged for 15 min at 12,000× *g* and the resulting pellet was washed in 70% ethanol, air dried for 10 min, and dissolved in DEPC-treated ddH_2_O. The quality and quantity of extracted RNA samples were analyzed by fiber optic spectrophotometry using a NanoDrop 2000c (Thermo Scientific, Waltham, MA, USA). While RNA quantity was measured using the A_260_ absorbance in DEPC-treated ddH_2_O, the RNA purity was monitored by A_260_/A_280_ absorbance ratios of sample dilutions in Tris pH 7.4. RNA samples with A_260_/A_280_ ratios of 1.8–2.1 were used for cDNA in the study. The average concentration of the RNA samples used in the study was 220 ± 132 ng/µL. Potential residual genomic DNA was eliminated with RNase-free DNase I digestion (Thermo Scientific). Complementary DNA (cDNA) was synthesized from 500-ng total DNase I treated RNA per reaction using the PrimScriptTM Reagent Kit (Takara Inc., Shiga, Japan) following the manufacturer’s instructions. Briefly, in a 10-µL reaction with 1× reaction buffer including MgCl_2_ inside an RNase-free tube, the appropriate volume of RNA sample was first treated with 0.5 µL of DNase I (1 U/µL) by gently mixing the reaction and incubating it at 37 °C for 30 min, followed by inactivation of DNase I with addition of 1 µL of 50 mM EDTA and incubation at 65 °C for 10 min. This DNase I treated RNA sample (11 µL) was then used for cDNA synthesis in a 20-µL reaction containing 4 µL of 5× PrimeScript^TM^ buffer, 1 µL of PrimeScript^TM^ RT Enzyme mix I, 1 µL of 50 µM Oligo dT primer, 1 µL of 100 µM Random 6 mers, and 2 µL of RNase-free ddH_2_O. The RT reaction was mixed well and incubated at 37 °C for 15 min, followed by inactivation of the RT enzyme at 85 °C for 5 s. The cDNA samples were kept at −20 °C for further use.

### 4.3. Candidate Reference Gene Selection

A total of 8 commonly used housekeeping genes with a wide range of biological functions were used in this study (see Table 2 for details). Six of the eight genes were picked from the internal control panel of genes offered by PrimerDesign Ltd. (Southampton, UK) with the following “anchor nucleotide” and “context sequence length” [39] information for the primer sets: *18s* *rRNA* (235, 99 bp), *GAPDH* (1087, 142 bp), *RPL13A* (727, 223 bp), *SDHA* (1032, 154 bp), *UBC* (452, 192 bp), and *YWHAZ* (2585, 150 bp). The remaining two genes included *ACTB*, and *TFRC* with the following forward and reverse primer sequences: ACTB_F, 5′-CGAGCACAGAGCCTCGCCTTTGCC-3′, ACTB_R, 5′-TGTCGACGACGAGCGCGGCGATAT-3′; and TFRC_F, 5′-ACCGGCACCATCAAGCT-3′, TFRC_R, 5′-TGATCACGCCAGACTTTGC-3′.

### 4.4. Quantitative Real-Time PCR

The mRNA levels of the potential reference genes were quantified using Micro Amp Optical 8-Cap Strips (Life Technology, Pittsburgh, PA, USA) with an ABI 7300 Real-Time PCR System (Applied Biosystems, Foster City, CA, USA). Each 20-µL reaction contained 10 µL of RealQ Plus 2× PCR Master Mix Green, High ROX TM (Ampliqon Inc., Odense, Denmark), 25 ng of cDNA, and 300 nM of forward and reverse primers. PCR amplifications were performed in triplicates with an initial enzyme activation step at 95 °C for 10 min, followed by 40 cycles of 95 °C for 15 s (template denaturation), and 60 °C for 1 min (annealing and data collection). No-template controls (NTC) were included in each run. The inter-run technical variation was calculated as SD for three HKGs *Cq* values for a fixed repeated cDNA sample dilutions: *ACTB*, ±0.13; *GAPDH*, ±0.15; *YWHAZ*, ±0.14.

### 4.5. Gene Expression Stability Analysis

Analysis of expression stability for the candidate reference genes was initially carried out by using two commonly used statistical algorithms, geNorm (qbasePLUS software v2.2, Biogazelle) [40] and NormFinder (an Excel Add-in, provided by Molecular Diagnostic Labrotory, Dept. of Molecular Medicine, Aarhus University Hospital, Skejby Sygehus, Denmark) [41], both of which are Visual Basic Applications for Microsoft Excel, in parallel. The raw *Cq* values in an Excel spread sheet were also fed into the BestKeeper applet (verison 1, http://www.gene-quantification.com/bestkeeper.html) [44] for additional analysis.

## 5. Conclusions

To our knowledge, the present study is the first systematic evaluation of candidate HKGs as internal controls for normalization of gene expression profiles in autistic children. Based on the analysis of eight commonly used HKGs by geNorm and NormFinder algorithms in parallel, a combination of the top two most stable candidate reference genes, *GAPDH* and *YWHAZ*, could be recommended for normalization of RT-qPCR analysis of salivary transcriptome in drug naïve non-syndromic autistic male children. We hope that the information presented here paves the way for: (1) systematic evaluation of reference genes in ASD studies under different experimental settings; and (2) using saliva as an alternative source of RNA, instead of the brain and blood, for transcriptome analysis in ASD research. As a readily-available and resourceful biofluid, saliva could open a noninvasive window to the complex molecular world of autism not only for diagnosis but also for surveillance of disease status and treatment.

## Figures and Tables

**Table 1:** Internal control genes used in ASD gene expression studies.

Reference	RNA/Protein Source	Reference Gene(s)	Age (Year)	N	Sex
Purcell et al. (2001) [12]	Brain tissue	ACTIN ^WB^	5–54	10	M/F ^1^
	Cerebellum	*GAPDH* ^RP^			
Fatemi et al. (2001) [13]	Brain tissue	ACTIN ^WB^	23.5 ± 4.8	5	M
	Cerebellum				
Araghi-Niknam et al. (2003) [14]	Brain tissue	ACTIN ^WB^	23.8 ± 4.9	5	M
	Cerebellum, Frontal Cortex				
Samaco et al. (2004) [15]	Brain tissue	*TFRC* and *ACTIN* ^IF^	2–32	13	M/F ^8^
	Frontal Cortex	*GAPDH*, *H1*, and *TFRC* ^FISH^			
Hu et al. (2006) [16]	LCL	*18s rRNA* ^qP^	6–16	10	M
Nishimura et al. (2007 [17])	LCL	*HPRT1* ^qP^	AGRE	42	M
Garbett et al. (2008) [18]	Brain tissue	*ACTB* ^MA−qP^	4–30	6	M/F ^2^
	Sup. temporal gyrus				
Gregg et al. (2008) [19]	Whole blood	*DDR1*, *RPL37A*, and	CHARGE	35	M/F ^6^
		*SMARC2* ^MA−qP^			
Enstrom et al. (2009) [20]	PBL, Natural killer cells	*DDR1*, *RPL37A*, and *SMARC2* ^MA−qP^	2.3–5.6	52	M
Hu et al. (2009a) [21]	LCL	*MDH1*, *ARF1*, *ACSL5* ^MA−qP^	12.3 ± 3.7	116	M
Hu et al. (2009b) [22]	LCL	*18s rRNA* ^MA−qP^	7.8 ± 3.4	20	NA
Sheikh et al. (2010) [23]	Brain tissue	ACTIN ^WB^	8.9 ± 3.2	9	M/F ^4^
	Frontal Cortex				
Malik et al. (2011) [24]	PBL	ACTIN ^WB, NB^	8.4 ± 0.3	6	NA
Kuwano et al. (2011) [25]	Whole blood	*GAPDH* and *HDAC1* ^MA−qP^	26.7 ± 5.5	21	M/F ^4^
Ghahramani Seno et al. (2011) [26]	LCL	*TMEM*32 ^qP^ *TUBB* ^WB^	2–18	20	M/F ^7^
Luo et al. (2012) [27]	LCL	*GAPDH* ^MA−qP^	SSC	42	NA
Chow et al. (2012) [28]	Brain tissue	*ACTB*, *TBP*, and	2–56	20	M
	Prefrontal Cortex	*RPL13A* ^MA−qP^			
Kong et al. (2012) [29]	Whole blood	*GAPDH* ^MA−qP^	8.2 ± 3.0	170	M
Griesi-Oliveira et al. (2012) [30]	Dental pulp stem cells	*GAPDH*, *HPRT1*, *SDHA*, and *HMBS* ^qP^	10	1	F
Anitha et al. (2012) [31]	Brain tissue	*GAPDH* ^WB^	8–29	8	M/F ^2^
	Anterior cingulated gyrus, Motor Cortex, thalamus	*B2M*, *HPRT1*, *RPL13A*, *GAPDH*, and *ACTB* ^qP^			
Ginsberg et al. (2012) [32]	Brain tissue	*GAPDH* ^qP^	2–60	9	M/F
	Occipital and cerebellar hemispheric cortices				
Choi et al. (2014) [33]	Brain Tissue	*GAPDH* ^qP^	4.5–82	29	M/F ^8^
	Cerebellum				
Nardone et al. (2014) [34]	Brain Tissue	*GAPDH*, *HPRT1*, *POLR2α*, and *SDHA* ^qP^	18–51	13	M/F ^2^
	Anterior cingulated gyrus				
	Prefrontal Cortex				

N, number of ASD samples; NA, not available; F ^N^, number of female ASD samples; Regular capitalized abbreviations under the Reference Gene(s) columns are indicative of proteins, whereas the italicized ones denote RNA or mRNA. Sample sources: AGRE, Autism Genetic Resource Exchange; CHARGE, Childhood Autism Risks from Genetics and Environment; SSC, Simon Simplex Collection. Cell lines: LCL, Lymphoblastoid Cell Line; PBL, Peripheral Blood Lymphocyte. Techniques: FISH, Fluorescence in situ Hybridization; IF, Immunofluorescence; MA-qP, Microarray-RT-qPCR; qP, Reverse transcriptase quantitative real-time PCR; NB, Northern Blot; RP, semi-quantitative RT-PCR; WB, Western Blot.

**Table 2:** List of the housekeeping genes evaluated as candidate reference genes during the first phase.

Gene Name	Function	Gene Symbol	mRNA Accession No.	Amplicon ^2^ Length (bp)
18S ribosomal RNA	Ribosomal RNA Subunit	*18s* *rRNA*	M10098 ^1^	99 *
β-actin	Cytoskeletal structural protein	*ACTB*	NM_001101	94
Glyceraldehyde-3-phosphate dehydrogenase	Glycolytic enzyme	*GAPDH*	NM_002046	142 *
Ribosomal protein L13a	Structural component of large subunit of Ribosome	*RPL13A*	NM_012423	223 *
Succinate dehydrogenase complex subunit A, flavoprotein	Electron transporter in the Krebs cycle	*SDHA*	NM_004168	154 *
Transferrin receptor	Cellular iron uptake	*TFRC*	NM_003234	134
Ubiquitin C	Protein degradation	*UBC*	NM_021009	192 *
Tyrosine 3 monooxygenase activation protein, zeta polypeptide	Signal transduction	*YWHAZ*	NM_003406	150 *

^1^ Non-coding RNA; ^2^ The numbers with asterisks given for *18s rRNA*, *GAPDH*, *RPL13A*, *SDHA*, *UBC*, and *YWHAZ* do not reflect exact Amplicon sizes. They are indicative of the “context sequence length” [39] information provided by PrimerDesign Ltd. (Southampton, UK).

**Table 3:** The mean *Cq* values (±SD, *n* = 2–4) of samples studied in phase I.

Gene Symbol	Control			Autism			Overall SD
	Mean	SD	N	Mean	SD	N	
*18s* *rRNA*	16.06	1.78	2	14.58	0.66	2	1.04
*ACTB*	25.87	0.38	2	29.18	1.77	2	2.34
*GAPDH*	26.27	0.29	2	27.01	0.44	2	0.52
*RPL13A*	31.09	0.89	2	32.72	NA	1	1.15
*SDHA*	33.73	NA	1	28.93	0.32	2	3.40
*TFRC*	31.14	1.73	2	33.22	1.58	2	1.47
*UBC*	30.22	0.62	2	32.23	0.59	2	1.42
*YWHAZ*	27.54	0.61	2	28.07	1.11	2	0.37

N, number of samples (from independent individuals) with valid signals; NA, not applicable; SD, standard deviation.

**Table 4:** Amplification efficiency data for candidate reference genes tested in phase II.

Gene Symbol	Slope	R^2^	Efficiency (%)
*ACTB*	−3.400	0.981	96.8
*GAPDH*	−3.303	0.989	100.9
*UBC*	−3.747	0.974	84.8
*YWHAZ*	−3.398	0.984	97.2

Standard curves were used to calculate the reaction efficiency for each gene, using a five-point serial dilution made from pooled stock cDNAs. The amplification efficiency percentage for each gene was calculated using the equation *E* = 10 ^(−1/Slope)^ − 1 multiplied by 100, where *E* stands for the efficiency and slope is the gradient of the best fit line. R^2^: linear regression coefficient.

## Supplementary Materials

Supplementary materials can be found at www.mdpi.com/1422-0067/17/10/1711/s1.

## Abbreviations

ACTB, β-actin protein; *ACTB*, β-actin gene; ASD, Autism spectrum disorders; Cq, Quantification Cycle; *GAPDH*, Glyceraldehyde-3-phosphate dehydrogenase gene; HKG, House Keeping Gene; MIQE, Minimum Information for Publication of Quantitative Real-Time PCR Experiments; RT-qPCR, Reverse Transcriptase Quantitative real-time PCR; *UBC*, Ubiquitin C gene; *YWHAZ*, Tyrosine 3 monooxygenase activation, zeta polypeptide gene.

## References

[1] Lai M.C., Lombardo M.V., Baron-Cohen S. (2014). Autism. Lancet.

[2] Lord C., Risi S., DiLavore P.S., Shulman C., Thurm A., Pickles A. (2006). Autism from 2 to 9 years of age. Arch. Gen. Psychiatry.

[3] Werling D.M., Geschwind D.H. (2013). Sex differences in autism spectrum disorders. Curr. Opin. Neurol..

[4] Betancur C., Coleman M., Joseph D., Buxbaum P.R.H. (2013). Etiological Heterogeneity in Autism Spectrum Disorders: Role of Rare Variants. The Neuroscience of Autism Spectrum Disorders.

[5] Ch’ng C., Kwok W., Rogic S., Pavlidis P. (2015). Meta-analysis of gene expression in autism spectrum disorder. Autism Res..

[6] Berg J.M., Geschwind D.H. (2012). Autism genetics: Searching for specificity and convergence. Genome Biol..

[7] Morey J.S., Ryan J.C., van Dolah F.M. (2006). Microarray validation: Factors influencing correlation between oligonucleotide microarrays and real-time PCR. Biol. Proced. Online.

[8] Nolan T., Hands R.E., Bustin S.A. (2006). Quantification of mrna using real-time RT-PCR. Nat. Protoc..

[9] Huggett J., Dheda K., Bustin S., Zumla A. (2005). Real-time RT-PCR normalisation; strategies and considerations. Genes Immun..

[10] Bustin S.A., Benes V., Garson J.A., Hellemans J., Huggett J., Kubista M., Mueller R., Nolan T., Pfaffl M.W., Shipley G.L. (2009). The MIQE guidelines: Minimum information for publication of quantitative real-time PCR experiments. Clin. Chem..

[11] Li R., Shen Y. (2013). An old method facing a new challenge: Re-visiting housekeeping proteins as internal reference control for neuroscience research. Life Sci..

[12] Purcell A.E., Jeon O.H., Zimmerman A.W., Blue M.E., Pevsner J. (2001). Postmortem brain abnormalities of the glutamate neurotransmitter system in autism. Neurology.

[13] Fatemi S.H., Stary J.M., Halt A.R., Realmuto G.R. (2001). Dysregulation of reelin and Bcl-2 proteins in autistic cerebellum. J. Autism Dev. Disord..

[14] Araghi-Niknam M., Fatemi S.H. (2003). Levels of Bcl-2 and p53 are altered in superior frontal and cerebellar cortices of autistic subjects. Cell. Mol. Neurobiol..

[15] Samaco R.C., Nagarajan R.P., Braunschweig D., LaSalle J.M. (2004). Multiple pathways regulate MeCP2 expression in normal brain development and exhibit defects in autism-spectrum disorders. Hum. Mol. Genet..

[16] Hu V.W., Frank B.C., Heine S., Lee N.H., Quackenbush J. (2006). Gene expression profiling of lymphoblastoid cell lines from monozygotic twins discordant in severity of autism reveals differential regulation of neurologically relevant genes. BMC Genom..

[17] Nishimura Y., Martin C.L., Vazquez-Lopez A., Spence S.J., Alvarez-Retuerto A.I., Sigman M., Steindler C., Pellegrini S., Schanen N.C., Warren S.T. (2007). Genome-wide expression profiling of lymphoblastoid cell lines distinguishes different forms of autism and reveals shared pathways. Hum. Mol. Genet..

[18] Garbett K., Ebert P.J., Mitchell A., Lintas C., Manzi B., Mirnics K., Persico A.M. (2008). Immune transcriptome alterations in the temporal cortex of subjects with autism. Neurobiol. Dis..

[19] Gregg J.P., Lit L., Baron C.A., Hertz-Picciotto I., Walker W., Davis R.A., Croen L.A., Ozonoff S., Hansen R., Pessah I.N. (2008). Gene expression changes in children with autism. Genomics.

[20] Enstrom A.M., Lit L., Onore C.E., Gregg J.P., Hansen R.L., Pessah I.N., Hertz-Picciotto I., van de Water J.A., Sharp F.R., Ashwood P. (2009). Altered gene expression and function of peripheral blood natural killer cells in children with autism. Brain Behav. Immun..

[21] Hu V.W., Sarachana T., Kim K.S., Nguyen A., Kulkarni S., Steinberg M.E., Luu T., Lai Y., Lee N.H. (2009). Gene expression profiling differentiates autism case–controls and phenotypic variants of autism spectrum disorders: Evidence for circadian rhythm dysfunction in severe autism. Autism Res..

[22] Hu V.W., Nguyen A., Kim K.S., Steinberg M.E., Sarachana T., Scully M.A., Soldin S.J., Luu T., Lee N.H. (2009). Gene expression profiling of lymphoblasts from autistic and nonaffected sib pairs: Altered pathways in neuronal development and steroid biosynthesis. PLoS ONE.

[23] Sheikh A., Li X., Wen G., Tauqeer Z., Brown W., Malik M. (2010). Cathepsin D and apoptosis related proteins are elevated in the brain of autistic subjects. Neuroscience.

[24] Malik M., Sheikh A.M., Wen G., Spivack W., Brown W.T., Li X. (2011). Expression of inflammatory cytokines, Bcl-2 and cathepsin D are altered in lymphoblasts of autistic subjects. Immunobiology.

[25] Kuwano Y., Kamio Y., Kawai T., Katsuura S., Inada N., Takaki A., Rokutan K. (2011). Autism-associated gene expression in peripheral leucocytes commonly observed between subjects with autism and healthy women having autistic children. PLoS ONE.

[26] Ghahramani Seno M.M., Hu P., Gwadry F.G., Pinto D., Marshall C.R., Casallo G., Scherer S.W. (2011). Gene and miRNA expression profiles in autism spectrum disorders. Brain Res..

[27] Luo R., Sanders S.J., Tian Y., Voineagu I., Huang N., Chu S.H., Klei L., Cai C., Ou J., Lowe J.K. (2012). Genome-wide transcriptome profiling reveals the functional impact of rare de novo and recurrent CNVs in autism spectrum disorders. Am. J. Hum. Genet..

[28] Chow M.L., Pramparo T., Winn M.E., Barnes C.C., Li H.-R., Weiss L., Fan J.-B., Murray S., April C., Belinson H. (2012). Age-dependent brain gene expression and copy number anomalies in autism suggest distinct pathological processes at young versus mature ages. PLoS Genet..

[29] Kong S.W., Collins C.D., Shimizu-Motohashi Y., Holm I.A., Campbell M.G., Lee I.-H., Brewster S.J., Hanson E., Harris H.K., Lowe K.R. (2012). Characteristics and predictive value of blood transcriptome signature in males with autism spectrum disorders. PLoS ONE.

[30] Griesi-Oliveira K., Moreira Dde P., Davis-Wright N., Sanders S., Mason C., Orabona G.M., Vadasz E., Bertola D.R., State M.W., Passos-Bueno M.R. (2012). A complex chromosomal rearrangement involving chromosomes 2, 5, and X in autism spectrum disorder. Am. J. Med. Genet. B Neuropsychiatr. Genet..

[31] Anitha A., Nakamura K., Thanseem I., Yamada K., Iwayama Y., Toyota T., Matsuzaki H., Miyachi T., Yamada S., Tsujii M. (2012). Brain region-specific altered expression and association of mitochondria-related genes in autism. Mol. Autism.

[32] Ginsberg M.R., Rubin R.A., Falcone T., Ting A.H., Natowicz M.R. (2012). Brain transcriptional and epigenetic associations with autism. PLoS ONE.

[33] Choi J., Ababon M.R., Soliman M., Lin Y., Brzustowicz L.M., Matteson P.G., Millonig J.H. (2014). Autism associated gene, engrailed2, and flanking gene levels are altered in post-mortem cerebellum. PLoS ONE.

[34] Nardone S., Sams D.S., Reuveni E., Getselter D., Oron O., Karpuj M., Elliott E. (2014). DNA methylation analysis of the autistic brain reveals multiple dysregulated biological pathways. Transl. Psychiatry.

[35] Pandit P., Cooper-White J., Punyadeera C. (2013). High-yield RNA-extraction method for saliva. Clin. Chem..

[36] Lee Y.H., Zhou H., Reiss J.K., Yan X., Zhang L., Chia D., Wong D.T. (2011). Direct saliva transcriptome analysis. Clin. Chem..

[37] Park N.J., Li Y., Yu T., Brinkman B.M., Wong D.T. (2006). Characterization of RNA in saliva. Clin. Chem..

[38] Seugnet L., Boero J., Gottschalk L., Duntley S.P., Shaw P.J. (2006). Identification of a biomarker for sleep drive in flies and humans. Proc. Natl. Acad. Sci. USA.

[39] Bustin S.A., Benes V., Garson J.A., Hellemans J., Huggett J., Kubista M., Mueller R., Nolan T., Pfaffl M.W., Shipley G.L. (2011). Primer sequence disclosure: A clarification of the MIQE guidelines. Clin. Chem..

[40] Vandesompele J., de Preter K., Pattyn F., Poppe B., van Roy N., de Paepe A., Speleman F. (2002). Accurate normalization of real-time quantitative RT-PCR data by geometric averaging of multiple internal control genes. Genome Biol..

[41] Andersen C.L., Jensen J.L., Orntoft T.F. (2004). Normalization of real-time quantitative reverse transcription-PCR data: A model-based variance estimation approach to identify genes suited for normalization, applied to bladder and colon cancer data sets. Cancer Res..

[42] Lallemant B., Evrard A., Combescure C., Chapuis H., Chambon G., Raynal C., Reynaud C., Sabra O., Joubert D., Hollande F. (2009). Reference gene selection for head and neck squamous cell carcinoma gene expression studies. BMC Mol. Biol..

[43] Livak K.J., Schmittgen T.D. (2001). Analysis of relative gene expression data using real-time quantitative PCR and the 2^−∆∆*C*t^ method. Methods.

[44] Pfaffl M.W., Tichopad A., Prgomet C., Neuvians T.P. (2004). Determination of stable housekeeping genes, differentially regulated target genes and sample integrity: Bestkeeper—Excel-based tool using pair-wise correlations. Biotechnol. Lett..

[45] Keller S., Sarchiapone M., Zarrilli F., Videtic A., Ferraro A., Carli V., Sacchetti S., Lembo F., Angiolillo A., Jovanovic N. (2010). Increased BDNF promoter methylation in the Wernicke area of suicide subjects. Arch. Gen. Psychiatry.

[46] Arion D., Sabatini M., Unger T., Pastor J., Alonso-Nanclares L., Ballesteros-Yanez I., Garcia Sola R., Munoz A., Mirnics K., DeFelipe J. (2006). Correlation of transcriptome profile with electrical activity in temporal lobe epilepsy. Neurobiol. Dis..

[47] Arion D., Unger T., Lewis D.A., Levitt P., Mirnics K. (2007). Molecular evidence for increased expression of genes related to immune and chaperone function in the prefrontal cortex in schizophrenia. Biol. Psychiatry.

[48] Zhang L., Xiao H., Wong D.T. (2009). Salivary biomarkers for clinical applications. Mol. Diagn. Ther..

[49] Saxena V., Yadev N., Juneja V., Singh A., Tiwari U., Santha B. (2013). Saliva: A miraculous biofluid for early detection of disease. J. Oral Health Community Dent..

[50] Zhang L., Xiao H., Karlan S., Zhou H., Gross J., Elashoff D., Akin D., Yan X., Chia D., Karlan B. (2010). Discovery and preclinical validation of salivary transcriptomic and proteomic biomarkers for the non-invasive detection of breast cancer. PLoS ONE.

[51] Nohesara S., Ghadirivasfi M., Mostafavi S., Eskandari M.R., Ahmadkhaniha H., Thiagalingam S., Abdolmaleky H.M. (2011). DNA hypomethylation of MB-COMT promoter in the DNA derived from saliva in schizophrenia and bipolar disorder. J. Psychiatr. Res..

[52] Rahbar M.H., Samms-Vaughan M., Ma J., Bressler J., Dickerson A.S., Hessabi M., Loveland K.A., Grove M.L., Shakespeare-Pellington S., Beecher C. (2015). Synergic effect of GSTP1 and blood manganese concentrations in autism spectrum disorder. Res. Autism Spectr. Disord..

[53] Hicks S.D., Ignacio C., Gentile K., Middleton F.A. (2016). Salivary mirna profiles identify children with autism spectrum disorder, correlate with adaptive behavior, and implicate asd candidate genes involved in neurodevelopment. BMC Pediatr..

[54] Zhang L., Farrell J.J., Zhou H., Elashoff D., Akin D., Park N.H., Chia D., Wong D.T. (2010). Salivary transcriptomic biomarkers for detection of resectable pancreatic cancer. Gastroenterology.

[55] Yu H.R., Kuo H.C., Huang H.C., Huang L.T., Tain Y.L., Chen C.C., Liang C.D., Sheen J.M., Lin I.C., Wu C.C. (2011). Glyceraldehyde-3-phosphate dehydrogenase is a reliable internal control in western blot analysis of leukocyte subpopulations from children. Anal. Biochem..

[56] Chen J., Sochivko D., Beck H., Marechal D., Wiestler O.D., Becker A.J. (2001). Activity-induced expression of common reference genes in individual cns neurons. Lab. Investig..

[57] Silberberg G., Baruch K., Navon R. (2009). Detection of stable reference genes for real-time PCR analysis in schizophrenia and bipolar disorder. Anal. Biochem..

[58] Penna I., Vella S., Gigoni A., Russo C., Cancedda R., Pagano A. (2011). Selection of candidate housekeeping genes for normalization in human postmortem brain samples. Int. J. Mol. Sci..

[59] Steinhausen H.C., Erdin A. (1992). Abnormal psychosocial situations and ICD-10 diagnoses in children and adolescents attending a psychiatric service. J. Child Psychol. Psychiatry.

[60] Lord C., Pickles A., McLennan J., Rutter M., Bregman J., Folstein S., Fombonne E., Leboyer M., Minshew N. (1997). Diagnosing autism: Analyses of data from the autism diagnostic interview. J. Autism Dev. Disord..

[61] Goodman R. (1997). The strengths and difficulties questionnaire: A research note. J. Child. Psychol. Psychiatry.

