# Supplementary material for: Selection of Suitable Reference Genes for Analysis of Salivary Transcriptome in Non-Syndromic Autistic Male Children
Source: Int J Mol Sci. 2016 Oct 12;17(10):1711. doi: 10.3390/ijms17101711 (PMC5085743; doi:10.3390/ijms17101711)
Supplement: Supplementary file 1 [file ijms-17-01711-s001.pdf]

Supplementary Materials: Selection of Suitable Reference Genes for Analysis of Salivary Transcriptome in Non-Syndromic Autistic Male Children

Yasin Panahi, Fahimeh Salasar Moghaddam, Zahra Ghasemi, Mandana Hadi Jafari, Reza Shervin Badv, Mohammad Reza Eskandari and Mehrdad Pedram

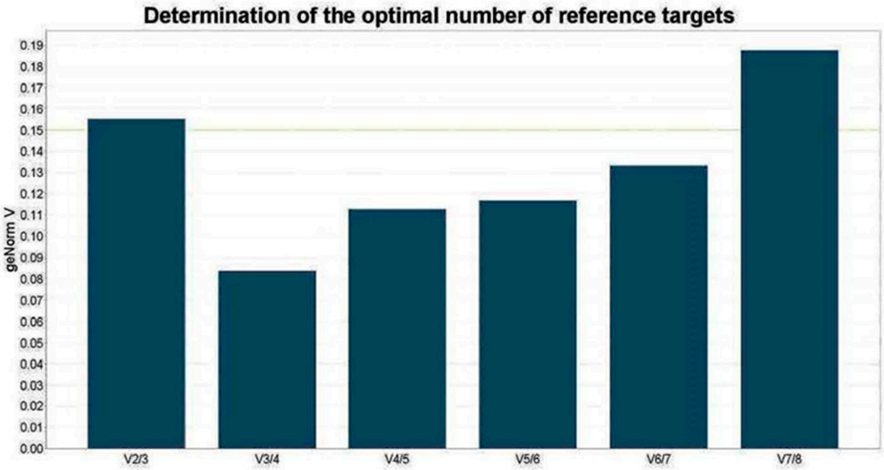

Figure S1. The optimal number of reference genes required for accurate normalization in phase I according to geNorm algorithm. Based on pairwise variation (V) by geNorm, the most stable three genes are required for normalization.

Table S1. Comparison of *ACTB Cq* values between adults and children (preliminary phase).

Samples	Age (Year)	C_q ± SD	Average	S_{tot}	2^{-Cq}	CV%
Ad_1	40	19.48 ± 0.31			1.36×10^{-6}	
Ad_2	40	19.46 ± 0.86			1.38×10^{-6}	
Ad_3	32	20.25 ± 0.38	19.54 ± 0.44	0.99	8.01×10^{-7}	28.5
Ad_4	45	19.01 ± 0.07			1.89×10^{-6}	
Ad_5	40	19.52 ± 0.03			1.33×10^{-6}	
Ch_1	5	24.72 ± 0.31			3.61×10^{-8}	
Ch_2	1	20.96 ± 0.61			4.90×10^{-7}	
Ch_3	9	27.48 ± 0.04	25.12 ± 2.60	0.69	5.34×10^{-9}	187.4
Ch_4	6	27.28 ± 0.01			6.13×10^{-9}	
Ch_5	3	25.19 ± 0.10			2.61×10^{-8}	

Ad, Adult; Ch, Children (Ch_1, represents an autistic patient, and $Ch_{2\text{–}5}$ healthy controls); SD, Standard deviation; S_{tot}, represents the sum of SD from replicate reactions calculated as sqrt $(SD_1^2 + SD_2^2 + \ldots + SD_N^2)$; CV, coefficient of variation for each group was calculated after linearization of the C_q values with 2^{-Cq}.

Table S2. The *Cq* values measured for samples studied in Phase 1.

Samples	*18s rRNA*		*ACTB*		*GAPDH*		*RPL13A*	
	Cq	SD	*Cq*	SD	*Cq*	SD	*Cq*	SD
A_1	15.15	0.01	27.67	0.30	27.35	0.04	32.72	0.61
A_2	14.02	0.01	30.69	0.35	26.67	0.33	ND	NA
C_1	14.64	0.35	25.93	0.56	26.09	0.27	30.35	0.03
C_2	17.48	0.29	25.80	0.01	26.45	0.21	31.84	0.36
**Samples**	***SDHA***		***TFRC***		***UBC***		***YWHAZ***	
	Cq	SD	*Cq*	SD	*Cq*	SD	*Cq*	SD
A_1	28.75	0.20	31.91	0.36	31.77	0.34	27.11	0.12
A_2	29.11	0.36	34.54	0.17	32.70	0.24	29.03	0.04
C_1	33.73	0.22	29.83	0.40	29.70	0.14	27.03	0.02
C_2	ND	NA	32.46	0.36	30.75	0.09	28.05	0.26

A, Autistic; C, Control; SD, standard deviation; ND, not detected; NA, not applicable. *Cq* values for each sample represent averages for respective triplicate reactions.

Table S3. The *Cq* values measured for all samples studied in Phase 2.

Samples	*ACTB*		*GAPDH*		*UBC*		*YWHAZ*	
	Cq	SD	*Cq*	SD	*Cq*	SD	*Cq*	SD
A_1	30.29	0.07	31.36	0.56	38.28	0.03	34.28	1.34
A_2	26.28	0.30	25.28	0.11	28.50	0.23	26.02	0.10
A_3	32.65	0.23	29.48	0.12	33.67	0.46	28.76	0.11
A_4	35.47	0.57	31.20	0.54	37.04	0.24	32.41	0.01
A_5	29.90	0.01	30.41	0.10	33.89	0.32	33.50	0.39
A_6	24.06	0.08	22.24	0.04	24.82	0.22	23.17	0.01
A_7	22.29	0.13	20.84	0.06	24.92	0.53	21.50	0.08
A_8	24.65	0.34	25.03	0.02	26.35	0.28	24.52	0.31
A_9	21.26	0.27	21.98	0.33	24.33	0.32	23.77	0.13
Average	27.43 ± 4.89	0.83	26.42 ± 4.24	0.87	30.20 ± 5.55	0.97	27.55 ± 4.84	1.45
C_1	22.56	0.16	21.33	0.02	23.41	0.04	22.41	0.12
C_2	27.52	0.35	28.00	0.01	31.66	0.42	29.32	0.04
C_3	30.18	0.07	30.41	0.14	34.30	0.22	30.63	0.07
C_4	26.38	0.25	27.40	0.02	30.88	0.17	28.65	0.04
C_5	23.34	0.21	23.68	0.28	28.95	0.43	24.58	0.23
C_6	24.01	0.49	24.63	0.31	28.94	0.13	24.67	0.28
C_7	27.48	0.38	28.62	0.21	39.28*	0.03	28.19	0.04
C_8	22.40	0.09	21.69	0.30	26.73	0.18	23.67	0.30
C_9	21.03	0.26	20.95	0.12	23.27	0.46	21.75	0.28
Average	24.99 ± 3.03	0.85	25.19 ± 3.53	0.59	28.52 ± 3.89	0.84	25.99 ± 4.89	0.57

A, Autistic; C, Control; SD, standard deviation. *Cq* values for each sample represent averages for respective triplicate reactions. The star in the *UBC* column indicates an undetected *Cq* value, which was replaced with an assigned value based on the maximum *Cq* detected for that assay plus 1 (Max *Cq* + 1). The rows labeled as Average show the mean *Cq* values (±SD, n = 9) for the corresponding gene in each group.

Table S4. Expression stability of the candidate HKGs in saliva samples evaluated by BestKeeper.

**Phase I: Eight Candidate HKGs; Four Samples**								
**Gene**	**Geo. Mean**	**Ar. Mean**	**Min (*Cq*)**	**Max (*Cq*)**	**SD**	**CV (%)**	**Coeff. of Corr. (r)** [1]	**Power of HKG**
18s rRNA	15.27	15.32	14.02	17.48	1.08 (4)	7.04	0.424	1.92
ACTB	27.45	27.52	25.80	30.69	1.66 (7)	6.02	0.404	2.55
GAPDH	26.64	26.64	26.09	27.35	0.37 (1)	1.39	0.365	1.22
RPL13A	32.56	32.61	30.35	35.54 [2]	1.52 (6)	4.65	0.661	4.34
SDHA	31.65	31.78	28.75	35.54 [2]	2.85 (8)	8.98	-0.107	0.69
TFRC	32.14	32.19	29.83	34.54	1.32 (5)	4.09	0.858	5.42
UBC	31.21	31.23	29.70	32.70	1.01 (3)	3.22	0.673	2.43
YWHAZ	27.79	27.81	27.03	29.03	0.74 (2)	2.64	0.272	1.30
**Phase II: Four Candidate Genes; 18 Samples**								
**Gene**	**Geo. Mean**	**Ar. Mean**	**Min (*Cq*)**	**Max (*Cq*)**	**SD**	**CV (%)**	**Coeff. of Corr. (r)** [3]	**Power of HKG**
ACTB	25.91	26.21	21.03	35.47	3.36 (1)	12.84	0.948 (4)	1.91 (2/3)
GAPDH	25.54	25.81	20.84	31.36	3.38 (2)	13.10	0.991 (1)	1.90 (1)
UBC	29.54	29.96	23.27	39.28	4.37 (4)	14.59	0.952 (3)	2.08 (4)
YWHAZ	26.48	26.77	21.50	34.28	3.51 (3)	13.12	0.968 (2)	1.91 (2/3)

Geo. mean, Geometric mean of minimum and maximum *Cq* values; Ar. mean, Arithmetic mean of minimum and maximum *Cq* values; Min *Cq*, minimum *Cq* value; Max *Cq*, maximum *Cq* value; SD, standard deviation; CV, coefficient of variation; Coeff. of Corr., coefficient of correlation. The numbers in parentheses indicate the ranking of the HKGs in the respective columns. [1] *p*-values for pair-wise correlations are not provided by BestKeeper algorithm for Phase I, which makes the ranking in the (r) column not applicable; [2] Indicates an undetected *Cq* value, which was replaced with an assigned value based on the maximum *Cq* detected plus 1 (Max *Cq* + 1); [3] Phase II pair-wise correlation *p*-values = 0.001 for all combinations.
